# Methodology for designing intrahospital transportation of patients with suspected infectious disease that limits infection spread risk in China

**DOI:** 10.3389/fpubh.2022.926872

**Published:** 2023-01-04

**Authors:** Yuan Guo, Yanchu Li, Yanjun Wang, Pengpeng Liang, Xiaoli He, Bingjie Yu, Fangyu Chen, Qianhui Zeng

**Affiliations:** ^1^West China School of Nursing Department, West China Hospital of Sichuan University, Chengdu, China; ^2^Head and Neck Oncology Department, West China Hospital of Sichuan University, Chengdu, China; ^3^Outpatient Department, West China Hospital of Sichuan University, Chengdu, China; ^4^School of Architecture, Southwest Jiaotong University, Chengdu, China

**Keywords:** intrahospital transportation, virtual transfer pipeline, virtual-barrier space, out-patient area, COVID-19

## Abstract

**Aims:**

The transport of patients suspected of having COVID-19 requires careful consideration. Using paths selected at random and not accounting for person flow along the path are risk factors for infection spread. Intrahospital transportation (IHT) protocols and guidelines should be used to help reduce the risk of secondary virus transmission during transport. This study aimed to propose optimal IHT for patients with an infectious disease presenting in an out-patient area.

**Design:**

The map of a West China Hospital was used. We also used field investigation findings and simulated person flow to establish pathway length and transportation time. We identified three optimum pathways and estimated safety boundary marks, including a patient transportation border (PTB) and safety transportation border (STB). Finally, IHT, PTB, and STP formed a virtual transport pipeline (VTP) and a traceable IHT management system, which can generate a virtual isolation space.

**Results:**

The three pathways met efficiency, accessibility, and by-stander flow criteria. No facility characteristic modification was required.

**Conclusions:**

Using virtual models to identify pathways through out-patient hospital areas may help reduce the risk of infection spread.

## Introduction

Globally, 11% of patients with coronavirus disease 2019 (COVID-19) required admission to an intensive care unit (ICU) and 18% developed acute respiratory distress syndrome, requiring admission to hospitals that provide higher levels of care (1, 2). Intrahospital transportation (IHT) is among the most frequently performed tasks involving hospitalized patients (3–5). Previous studies have proposed various IHT strategies, focusing on transportation methods that help reduce infection risks. Risk management in this context requires that the transport is well-organized, efficient, and accompanied by suitable monitoring, equipment, and personnel (6), and that applicable protocols and guidelines are followed (7, 8). These protocols should account for personnel and by-stander safety.

Previous studies have shown that suitably equipped teams can ensure IHT that is safe to both patient and team (1, 9); however, these studies tended to focus on enclosed and well-equipped areas such as an ICU and neglected out-patient areas, which include open environments and a flow of by-standers and personnel that increase infection risk, making patient transport challenging (9).

Herein, we aimed to examine the safety of IHT for patients suspected of having COVID-19 in out-patient areas. In the clinical practice, although the personnel and equipment involved in IHT are planned, the path tends to be chosen at random, thus increasing the risk of infection spread among the people present along the path.

This study aimed to develop an IHT protocol for patients suspected of having COVID-19, focusing on limiting the risk of infection spread. We used the West China Hospital as a simulation model. The site is a university-affiliated medical center and contains almost 4,000 beds with an annual out-patient load of 5 million. The team included physicians, nurses, and engineers, tasked with evaluating the safety issues surrounding the IHT of patients suspected of having COVID-19. Having identified an optimal IHT pathway, we designed a virtual transport pipeline (VTP) network to generate a virtual path that contains an isolation space, forming a traceable IHT management system. VTP-based IHT may help improve patient and personnel safety, particularly in out-patient areas.

## Methods

The structure of a hospital is complex, making the development of evacuation plans challenging. Conducting evacuation drills helps test the applicable strategies; however, drills are difficult to conduct in a healthcare context. Thus, evacuation simulations are more feasible and may help improve safety. Models such as Building-Exodus and Pathfinder can help simulate hospital evacuation scenarios (10, 11). In this study, to define the virtual space and barrier parameters, we developed a VTP network. Thermodynamic analysis in Pathfinder (Version 2019.1.0508 x64, Thunderhead Engineering Inc. USA) was used to identify the shortest path distance and minimum human flow during transportation (12). Parameters including time, distance, access ways, and people flow were considered in the simulation. Based on the optimal VTP, we defined the patient transportation border (PTB) and safety transportation border (STB), which represented safety boundaries.

## Results

### Transportation time

**Figure 1:**
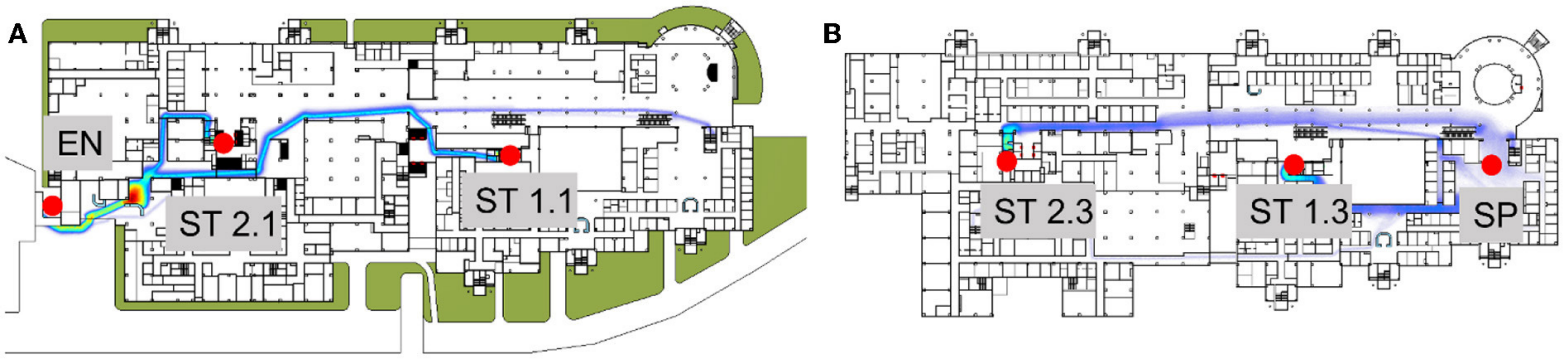
Transportation time from an outpatient to an isolation area with no limitations. According to the simulation, from the start point, patients could be transported though SP–ST1.3–ST1.1–EN or SP–ST2.3–ST2.1–EN. **(A)** First floor; **(B)** Third floor. EN, end position/Isolation area; SP, Start point.

We investigated whether the fastest pathway was the best pathway by using simulation software (parameters: non-interference situation, corridor width, and elevator size) (Figure 1). According to the simulation, 20 persons were placed at the start position (SP) on the third floor (respiratory clinic area); the remaining areas had no persons, including the end position (EN) on the first floor (isolation area). Two optimal paths were found. The two paths involved the use of two staircases (ST1.1/1.3 and ST2.1/2.3). The average time from the SP to the EN was approximately 150–204 s. However, these paths were not isolated from other paths in the considered areas; consequently, the number of potential contacts during IHT remained uncontrollable. Transportation time is only one parameter that should be considered in this context. Other parameters such as the optimal path, should be accounted for in future simulations.

### Pathway analysis

**Figure 2:**
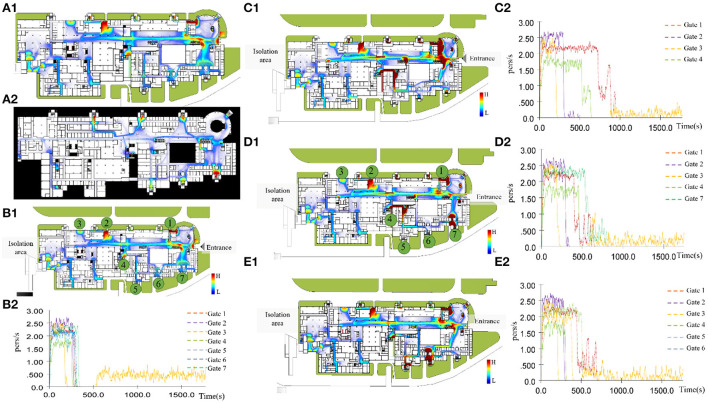
Simulation of flow distribution on the first and third floors, under daily conditions. **(A)** The main entrances, lobby corridor, elevators, and exits had a relatively dense flow distribution; **(B)** All entrances and exits open; **(C)** Entrances no. 5/6/7 and exits closed; **(D)** Entrances no. 5/6 closed and entrance no. 7 open; **(E)** Entrances no. 5/6 open and entrance no. 7 closed.

Flow distributions on the first and third floors were simulated. The main entrances, lobby corridor, elevators, and exits had a relatively dense flow distribution (Figure 2A). This simulation supports route selection, based on the likely number of contacts along the path.

To simulate scenarios that approximate real-world situations, we included data on the opening/closing of entrances/exits, to understand their impact on crowd evacuation (Figures 2B–E). Firstly, evacuations were quick when all entrances and exits were open (Figure 2B). Secondly, closing gates 5, 6, and 7 created some congestion points, particularly at gates 1, 2, and 4 (Figure 2C). Furthermore, the evacuation capacity of gate 7 was comparable to those of gates 5 and 6 (Figures 2D,E). Keeping gate 7 rather than gate 1 open was more beneficial for easing congestion, followed by gates 4 and 2. These findings suggest that gate 7 has a greater evacuation capacity than the other gates and that it may be useful during an evacuation and a trans-shipment. In addition, these findings indicate that, given the random nature of human flow in out-patient areas, protocols, design, and opening/closing relevant access points may improve the evacuation efficiency.

### VTP pathway design

**Figure 3:**
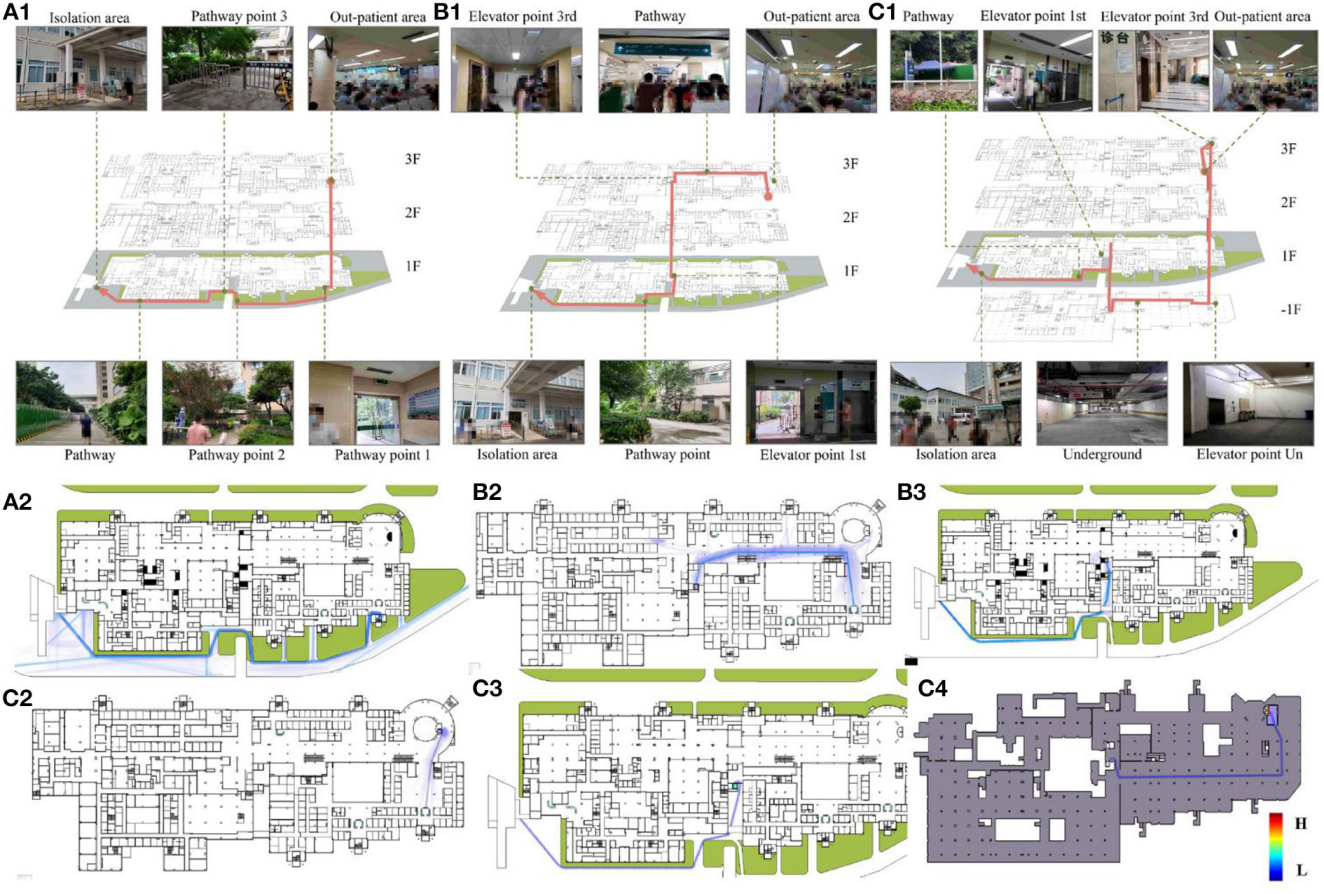
Three candidate pathways were designed, and transportation time and flow rate were simulated per pathway. Path no. 1 was accessible by stairs and an outdoor walkway **(A1)**; the time required to move from SP to EN was 190–263 s and the number of potential contacts was in the range 85–115 **(A2)**. Path no. 2 was accessible from an indoor walkway, elevator, and outdoor walkway **(B1)**; the time required to move from SP to EN was 220–296 s and the number of potential contacts was in the range 150–200 **(B2,B3)**. Path no. 3 was accessible by elevator E1, basement, elevator E2, and an outdoor walkway **(C1)**; the time required to move from SP to EN was 285–390 s and the number of potential contacts was in the range 80–110 **(C2–C4)**.

To investigate risk distribution differences among the different pathways, we considered field investigation findings, corridor width, and elevator size, arriving at three candidate paths (Figures 3A1–C1):

- Pathway no. 1 began near the stairs at the SP, giving the patient stair access from the third to the first floor; the patient arrived at the EN *via* an outdoor walkway. This path included mostly outdoor areas and a short indoor path.
- Pathway no. 2 began near an indoor walkway at the SP, giving the patient access to elevator E2 in the middle of a third-floor outpatient area, and stair access from the third to first floor; finally, the patient continued on an outdoor walkway to the EN. Approximately 50% of this path was inside the hospital.
- Pathway no. 3 began near the stairs at the SP, giving the patient access to elevator E1 near the SP, leading from the third floor to the basement; then, elevator E2 led from the basement to the first floor, where the patient walked down an outdoor walkway to the EN. Most of this path went through an underground parking lot.

During the simulations, we obtained transportation times and flow rates for all three paths, and placed five by-standers at the SP. The times required to move from the SP to the EN were 190–263 s, 220–296 s, and 285–390s for paths 1, 2, and 3, respectively, including the number of potential contacts in the ranges 85–115, 150–200, and 80–110, respectively (Figures 3A2,B2,B3,C2–C4).

### Safety distance boundary

**Figure 4:**
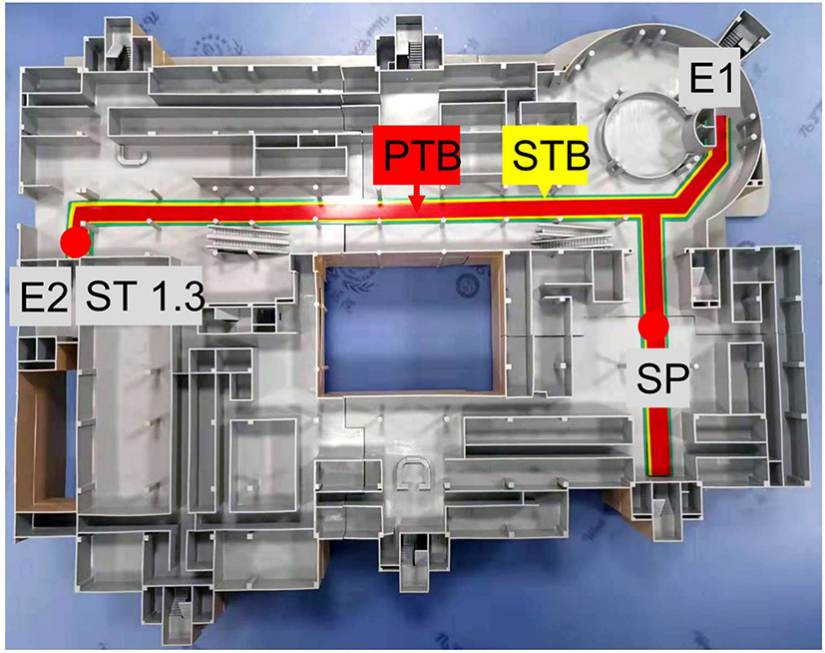
PTB and STB for the IHT model. A transportation pathway for a patient and a team member is marked in red. By-standers should be restricted to the yellow area.

We also developed a contingency plan. To establish a virtual parclose, a safety distance boundary was designed along the VTP pathway, which was used to standardize the PTB (red) and STB (yellow) (Figure 4). When the contingency plan was activated, the COVID-19 patient was transported along the PTB and by-standers were asked to keep away from the STB. The distance between the PTB and STB was 50.0 cm on each side.

In this study, different routes were identified, each with advantages and disadvantages. Based on efficiency, accessibility, and the number of potential contacts, path no. 2 emerged as the superior path in this simulation, followed by path no. 1.

## Discussion

IHT refers to moving a patient from one location to another within a hospital, which presents a complex logistical challenge (13). Any IHT involves six critical elements: transfer initiation request; transfer management request and information exchange; updates between transfer acceptance and patient transport; transport; patient admission and information availability; and measurement, evaluation, and feedback (14). In this study, we examined safe practices for IHT.

We estimated the number of potential COVID-19 patient contacts during IHT within an out-patient area. IHT is important during an infectious disease outbreak; applicable guidance requires continuous updates (15, 16) as patients may be diagnosed in an out-patient area and require admission to an ICU. A suitable IHT system helps control and prevent infection spread. However, previous studies have focused on IHT within the ICU (17, 18), reporting that adverse events during critical care transport were rare. The in-hospital mortality rate was 25%, with an extubation rate of 33.5% (19). Few previous studies have evaluated IHT for suspected COVID-19 patients (20, 21). McPherson reported that, providing that protective equipment and transport guidelines are properly used, children with COVID-19 can be transported safely with a low risk of adverse events for the patient and infection spread (20).

In contrast to previous studies, this study examined IHT in an out-patient area, which is an open environment with a large flow of people. Unlike the incidence rates for falls (22), drug dispensing errors (23), and unintended tube removal (24), those for IHT-related safety events outcomes remain unclear. Tangkulpanich proposed that hospital personnel are at an increased risk of infection, particularly during IHT, which involves close contact in a confined space without good ventilation, highlighting the need for personal protective equipment use. In addition, IHT through out-patient areas involves randomly chosen paths and many potential contacts, increasing the risk of infection spread.

The transport of patients with COVID-19 is complex and extends beyond isolation, containment, and disinfection. At the time of writing, IHT protocols for this patient group are not standardized, and recommendations for staff, perimeter size, and path traceability are unclear, resulting in an increased risk of poor outcomes. This study aimed to provide a solution for those scenarios, including a method of identifying the shortest path required, helping save time, improve traceability, and reduce applicable risks.

This study generated a virtual pipeline, which helps develop and adapt emergency transfer protocols that remain in place during outbreaks. Hospitals tend to require rapid responsiveness, which can be achieved by using the proposed model, helping reduce the risk of infection spread and improve IHT efficiency. This project presented an innovative VTP scheme. VTP is both a transfer process optimization and technology integration approach, which helps evaluate non-standardized behavior during outpatient transport, providing data-based support throughout the process; thus, the generated data may help in further IHT protocol development and in the continuous improvement of patient care.

This study had some limitations. Firstly, this was a single-center study based on an outpatient department of a West China Hospital; the VTP map was limited. These protocols may not apply to other hospitals, which may require tailored approaches. The presented models should be validated at other institutions before they can be generalized. Secondly, this study was based on a field investigation, and considered facility characteristics including elevator size and corridor width; considering patient type alone may not be enough in this context. Thus, future studies should include more extensive pathway simulations. This study presented a method for designing and evaluating IHT protocols in out-patient areas. Investigators adapting this methodology should consider applicable facility characteristics and people flow, among other variables. The presented methodology helps reduce infection spread during the IHT of patients with an infectious disease.

## Conclusions

This study has shown that IHT pathways for suspected COVID-19 patients should be planned rather than chosen at random. Moreover, time efficiency is not the only parameter that should be considered in an evacuation; human flow and access ways should be accounted for. Finally, using established pathways for IHT may help reduce the risk of infection spread.

Future studies should consider combining VTPs with cloud-based data to improve tracking and path recording. In addition, in the future, we intend to implement the presented protocol in a more complex setting to verify its applicability in improving patient safety.

## Data availability statement

The original contributions presented in the study are included in the article/supplementary material, further inquiries can be directed to the corresponding author.

## Author contributions

YL contributed to the design of the study, participated in data collection and analysis, and participated in drafting this article. YG oversaw data collection, wrote the statistical analysis plan, conducted the analysis, and drafted and revised the article. PL, QZ, BY, and FC assisted in conducting the study and participated in data collection and analysis. YW assisted in conducting the study and participated in data collection.
